# Advanced colorectal carcinoma with testicular metastasis in an adolescent: a case report

**DOI:** 10.1186/s13256-018-1831-8

**Published:** 2018-10-11

**Authors:** Adarsh Pratap Singh, Amit Kumar, Anita Dhar, Shipra Agarwal, Sudhir Bhimaniya

**Affiliations:** 1 Department of Surgery, AIIMS, New Delhi, India; 2 Department of Pathology, AIIMS, New Delhi, India; 3 Department of Cardiovascular Radiology, AIIMS, New Delhi, India

**Keywords:** Adolescent, Colorectal carcinoma, Testicular metastasis, Intestinal obstruction, Signet ring cell adenocarcinoma, Poor prognosis

## Abstract

**Background:**

Colorectal carcinoma in the pediatric age group is rare and tends to be very aggressive and present late, due to which it has a very poor prognosis. It may present with distant metastasis; however, metastasis to the testes is very rare and signifies an advanced stage of the disease. Surgery is the only effective modality to cure patients with localized colorectal carcinomas. However, statistics show a higher incidence of unresectable disease and a higher metastasis rate in childhood colorectal carcinomas. We present a case of advanced colorectal carcinoma with testicular metastasis in an adolescent.

**Case presentation:**

A 15-year-old Indian Hindu boy presented to surgical emergency with signs and symptoms of intestinal obstruction. He also had a history of passing blood and mucus per rectum. On examination he had abdominal distension. On digital rectal examination, a circumferential proliferative growth was felt 1 cm above the anal verge. On scrotal examination, a small nodule was felt in his right testis. In view of intestinal obstruction, he was taken into our emergency operation theater and a diverting loop sigmoid colostomy was performed to relieve the obstruction. A punch biopsy from anorectal growth was taken which suggested signet ring cell adenocarcinoma. Contrast-enhanced computed tomography of his chest, abdomen, and pelvis showed advanced colorectal carcinoma with distant metastasis. Ultrasonography of his testes showed a hypoechoic nodule in the right testis from which a needle aspiration biopsy was done which revealed metastatic adenocarcinoma.

**Conclusions:**

Childhood colorectal carcinomas have a very poor prognosis due to their aggressive nature and late presentation. In spite of all the advances in diagnosis and treatments, the overall long-term survival is still dismal in these patients. Due to the rarity of this disease, screening is not recommended for individuals under the age of 50. Thus, to improve outcome, early diagnosis and treatment is paramount. For that to happen, awareness needs to be created regarding pediatric colorectal carcinoma and its signs and symptoms.

## Background

Colorectal carcinoma (CRC) is the second most common alimentary tract carcinoma after liver tumors in children with an incidence of 1.3–2 cases per million; it is mostly present in the second decade of life [1–4]. It tends to be very aggressive and present late, due to which it has a very poor prognosis. It may present with distant metastasis; however, metastasis to the testes is very rare and signifies an advanced stage of the disease. Surgery is the only effective modality to cure patients with localized CRCs. We present a case of advanced CRC in an adolescent with testicular metastasis. This case has a rare presentation: it shows that CRC in an adolescent patient can present with testicular metastasis as well.

## Case presentation

A 15-year-old Indian Hindu boy from a low socioeconomic stratum presented to Surgical emergency with complaints of difficulty in passing stools for 1 month, passage of blood and mucus per rectum for 15 days, abdominal distension for 1 week, and obstipation for 3 days. These symptoms were associated with significant appetite and weight loss but there was no history of fever, jaundice, melena, hematemesis, hemoptysis, cough, chest pain, or shortness of breath. There was no history of similar illness or other malignancy in his family. He was not on any medication.

On examination he was conscious and oriented. He had a thin build and pallor. He was afebrile. His pulse rate was 84/minute and blood pressure (BP) was 110/74 mmHg. His abdomen was distended with no local bulge. On digital rectal examination, a circumferential proliferative growth was felt 1 cm above the anal verge, which was almost completely occluding the lumen. On scrotal examination, a small nodule was felt in his right testis. The rest of the systemic examination was normal.

**Fig. 1:**
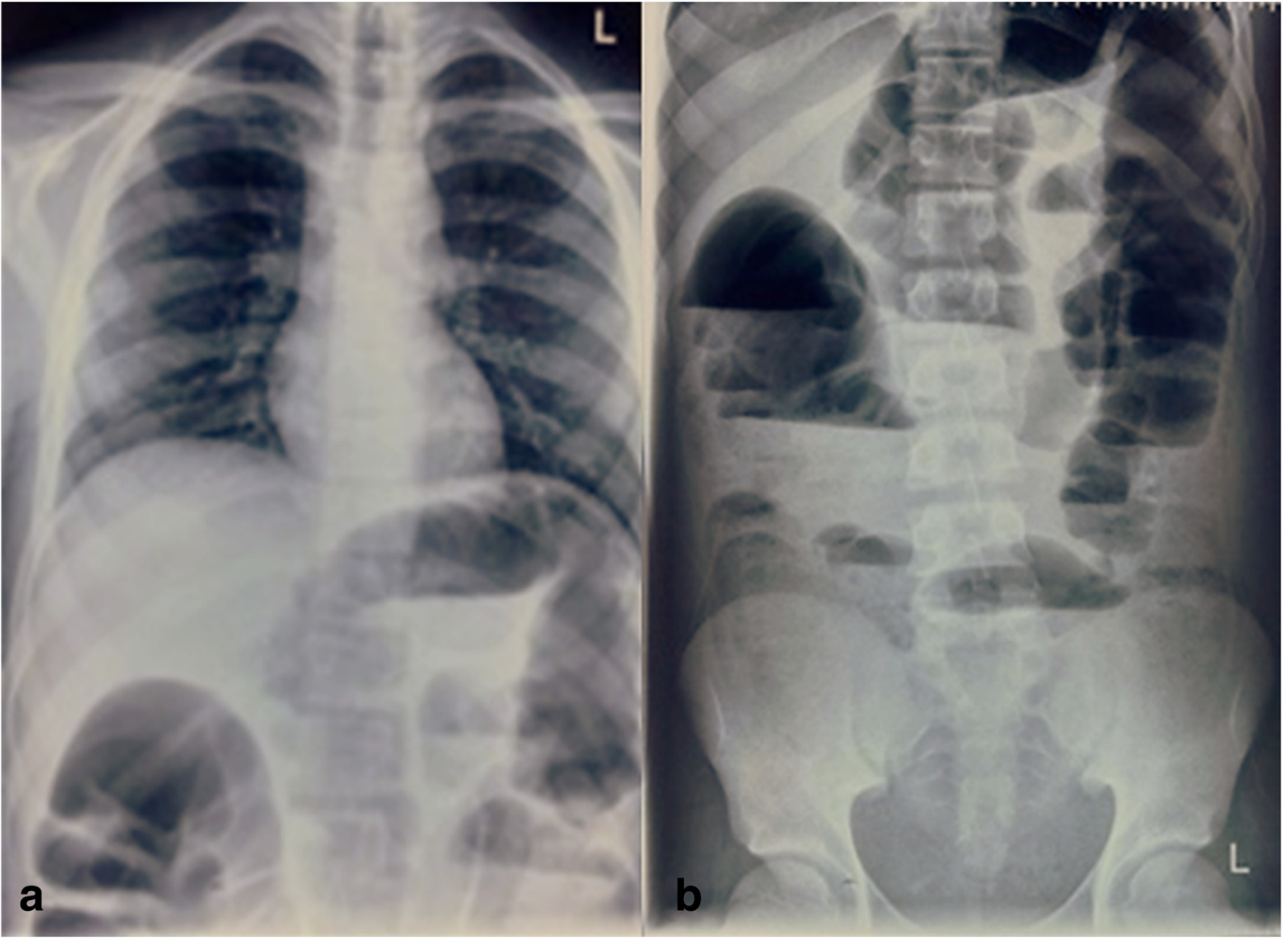
**a** Chest X-ray posteroanterior view; **b** abdominal X-ray anteroposterior erect view

An abdominal and chest X-ray was done as preliminary investigation which revealed signs of intestinal obstruction (Fig. 1).

In view of intestinal obstruction, he was taken into our emergency operation theater and a diverting loop sigmoid colostomy was performed. His symptoms were relieved, and stoma was well functioning and healthy.

**Fig. 2:**
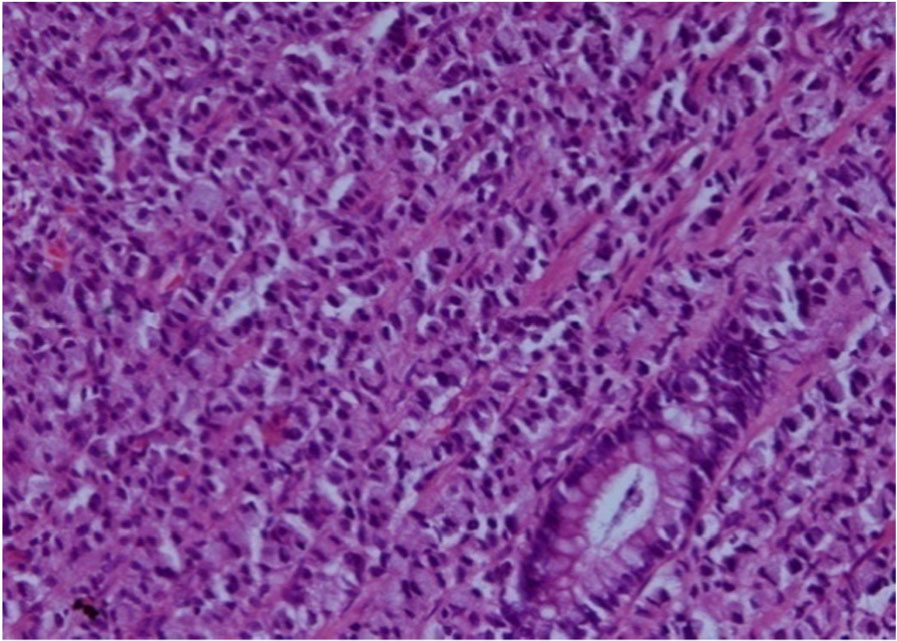
Histopathology. Rectal biopsy; hematoxylin and eosin × 200

A punch biopsy was taken from anorectal growth. The histopathological examination (HPE) report suggested signet ring cell adenocarcinoma of rectum (Fig. 2). Tumor markers report showed: carcinoembryonic antigen (CEA) 499.93, alpha-fetoprotein (AFP) 2.42, beta human chorionic gonadotropin (HCG) < 1.2, and lactate dehydrogenase (LDH) 593.

Routine investigations including complete blood count (CBC), and liver and renal function tests were within normal limits. His urine analysis was also normal.

**Fig. 3:**
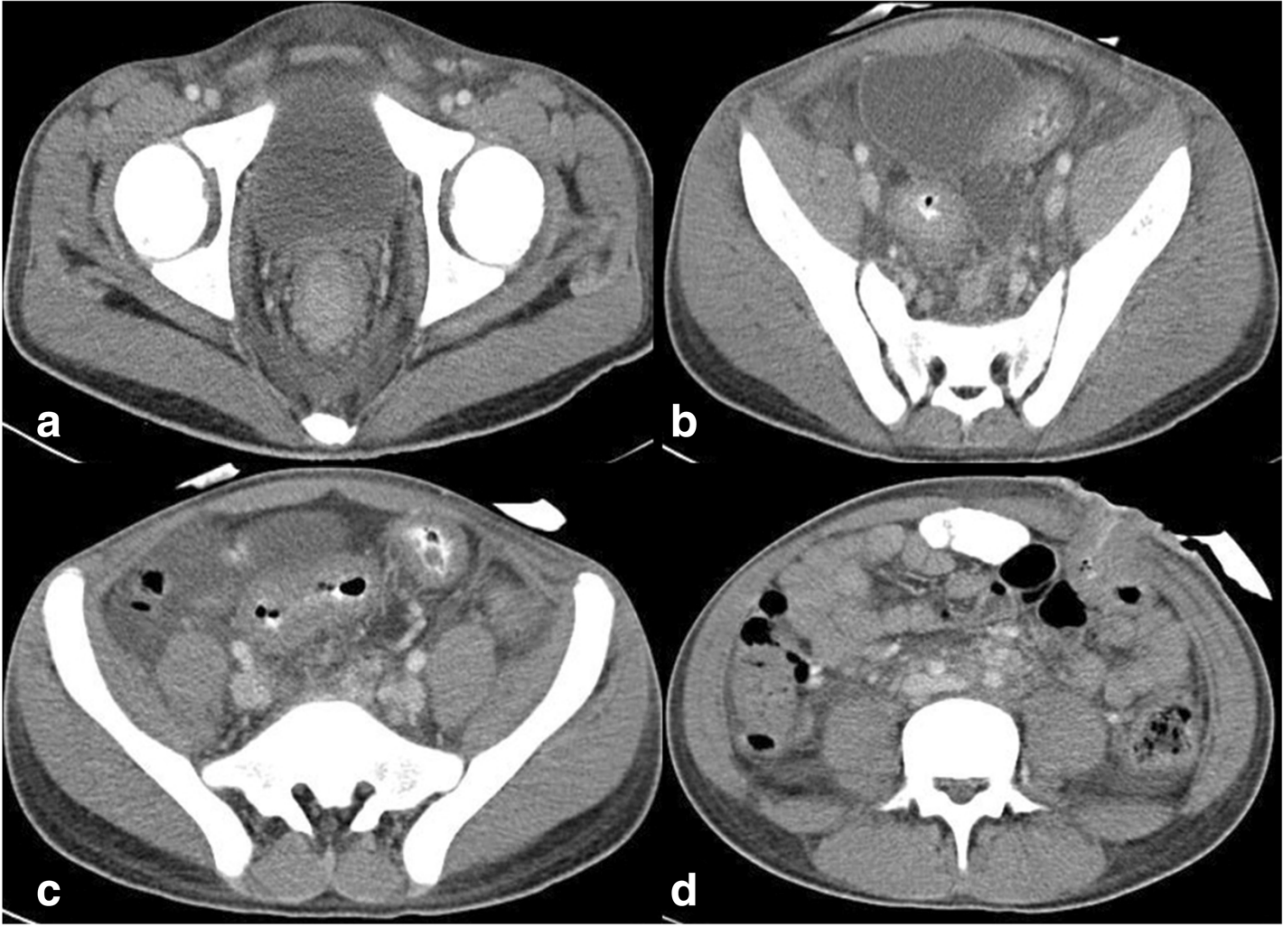
Contrast-enhanced computed tomography of the abdomen and pelvis. **a**–**c** Circumferential growth involving rectum and sigmoid colon. **d** Colostomy site

**Fig. 4:**
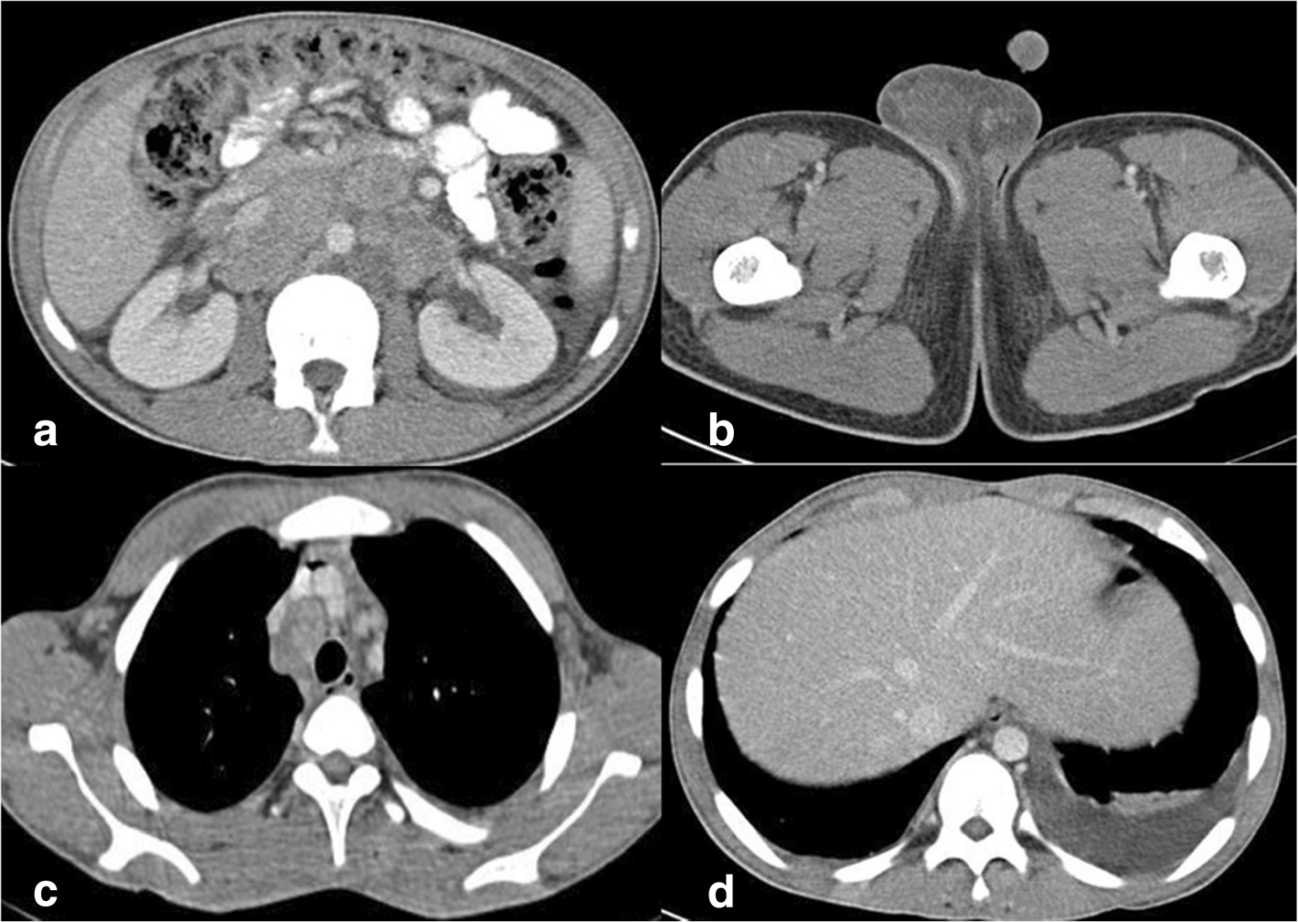
Contrast-enhanced computed tomography of the abdomen, pelvis, and chest. **a** Retroperitoneal lymphadenopathy. **b** Right testicular enlargement. **c** Mediastinal lymphadenopathy. **d** Left pleural effusion

**Fig. 5:**
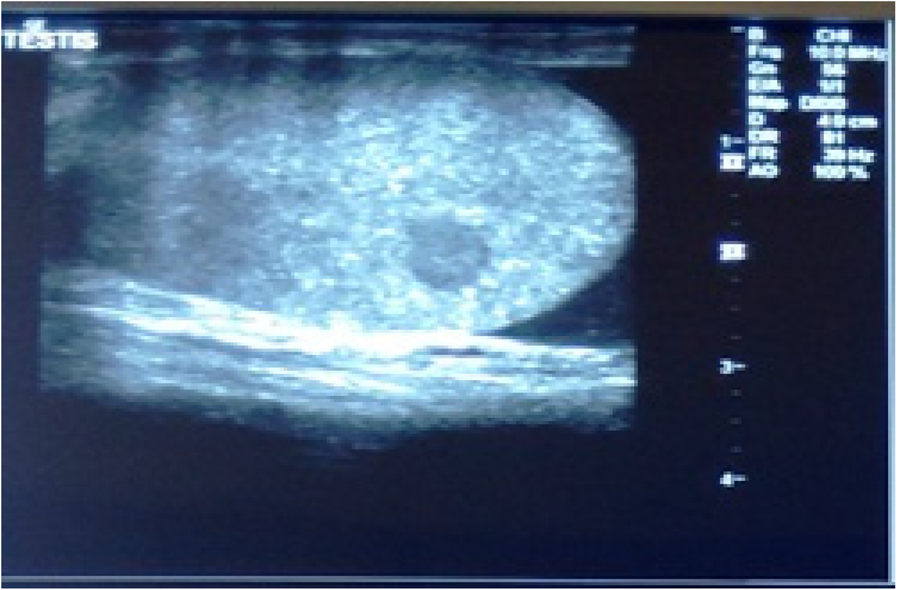
Ultrasonography of right testes showing nodule

**Fig. 6:**
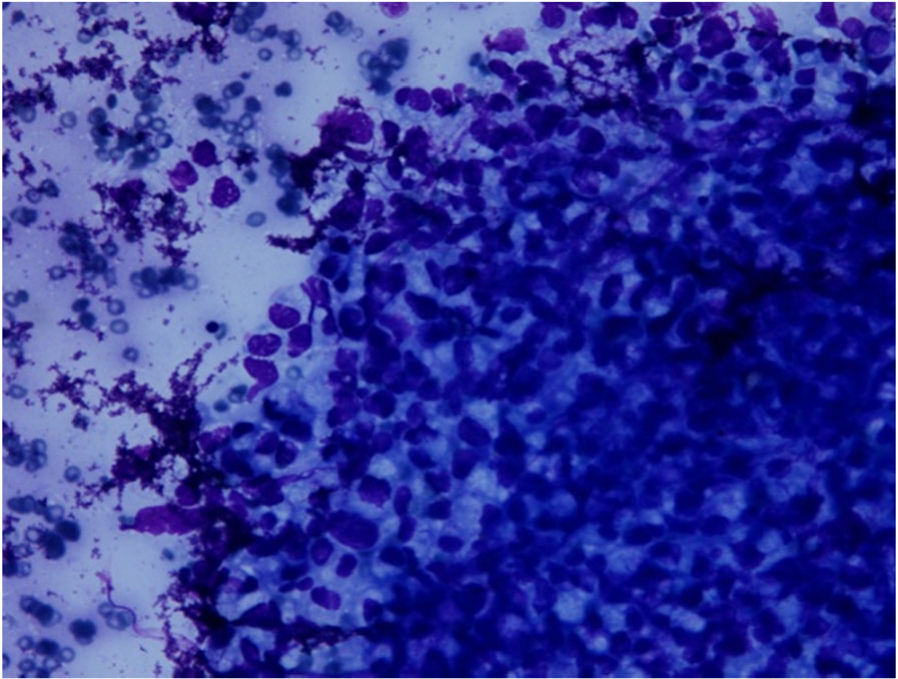
Histopathology. Fine-needle biopsy of right testes nodule; May–Grünwald–Giemsa × 200

Contrast-enhanced computed tomography (CECT) of his chest, abdomen, pelvis, and brain was done as a part of metastatic workup which showed diffuse circumferential homogenous thickening involving rectum approximately 1 cm from the anal verge and extending into sigmoid colon proximally up to colostomy site. Multiple enlarged lymph nodes, some showing necrosis, were noted in perirectal, iliac, bilateral para-aortic, periportal, and celiac regions. Moreover, multiple enlarged lymph nodes were seen in the mediastinum in bilateral paratracheal, prevascular, and subcarinal regions, and in left supraclavicular region. In addition, hepatomegaly with liver measuring 17.8 cm was present. However, no lesion was seen in liver parenchyma. There was mild left-sided pleural effusion (Figs. 3 and 4). There was no lesion in his brain suggestive of metastasis. His right testis was enlarged. Ultrasonography (USG) showed a hypoechoic nodule (Fig. 5) in his right testis from which a fine-needle aspiration biopsy was done, which revealed metastatic adenocarcinoma (Fig. 6).

In view of distant metastasis, we planned to give our patient neoadjuvant chemotherapy. However, within 2 weeks of surgery he developed progressive respiratory distress. A chest X-ray showed infiltrations and bilateral pleural effusion. He was intubated and was kept in our intensive care unit (ICU). However, his condition deteriorated and he developed multiple organ dysfunction syndrome (MODS) in the next few days. Eventually he died with multiple organ failure. An autopsy was not performed as per the wish of his family members. The entire course of illness from appearance of first symptom to death was only 2 months.

## Discussion

CRC in adolescent patients is rare. It usually presents with hematochezia/melena, abdominal pain, altered bowel habits, or with obstruction or perforation. This disease entity usually metastasizes to regional lymph nodes, liver, lungs, and peritoneum; metastasis to a testis is very rare [5] and very few cases have been reported in the literature. This case is unusual in the sense that our patient is an adolescent and had metastatic testicular nodule on presentation. This disease entity represents a very aggressive form of CRC.

Globally, CRC is the third most commonly diagnosed cancer in males and the second in females, with 1.4 million new cases and almost 694,000 deaths estimated to have occurred in 2012 [6]. Age is a major risk factor for sporadic CRC. It is generally considered to be a disease of the elderly, with more than 90% of patients with CRC being above 55 years of age. It rarely occurs in teenagers and adolescents [7].

More recent data from the US Surveillance, Epidemiology, and End Results (SEER) database and other Western cancer registries (http://www.cancervic.org.au/downloads/cec/cancer-in-vic/CCV-statistics-trends.pdf) suggest that CRC incidence is increasing in the under-50 age group while it is decreasing in older groups [8, 9]. These increases are predominantly left-sided cancers in general and rectal cancer in particular [10]. At present, screening is not recommended for individuals under the age of 50 unless they have a positive family history or a predisposing inherited syndrome.

Risk factors associated with young patients are inflammatory bowel disease, hereditary nonpolyposis colorectal cancer (HNPCC), and polyposis syndromes of gastrointestinal tract. *APC* gene mutation produces classic or attenuated familial polyposis coli [11]. Mutations in mismatch repair (*MMR*) genes, principally *MSH2* and *MLH1* but also *PMS1* and *PMS2*, cause HNPCC [12].

Data from the SEER program [13] suggest that the natural history of CRC in patients of ages 15 to 29 years is similar to that of older patients. However, current literature suggests that over 86% of those diagnosed under the age of 50 are symptomatic at diagnosis, and this is associated with a more advanced stage at diagnosis and poorer outcomes [11]. Approximately 60–86% of pediatric and adolescent patients have Dukes stage C or D [14]. The increased frequency of mucinous variants and preponderance of right-sided lesions contribute to the advanced stage at diagnosis [15, 16].

Typical symptoms/signs associated with CRC include hematochezia or melena, abdominal pain, otherwise unexplained iron deficiency anemia, and/or a change in bowel habits [17]. The patient may sometimes present with obstruction or perforation which carry a poor prognosis, independent of stage [18].

Patients may also present with signs/symptoms of metastatic disease. CRC can spread by lymphatic and hematogenous dissemination, as well as by contiguous and transperitoneal routes. The most common metastatic sites are the regional lymph nodes, liver, lungs, and peritoneum. Patients may present with signs or symptoms referable to any of these areas. The presence of right upper quadrant pain, abdominal distension, early satiety, supraclavicular adenopathy, or periumbilical nodules usually signals advanced, often metastatic, disease.

Metastatic carcinoma to the testis is rare [5] and most often incidentally found on autopsy. The most common tumors to metastasize to the testis are prostate (35%), lung (18%), melanoma (18%), kidney (9%) [9], and colorectal less than 8% [19].

There are less than 25 reported cases of colorectal cancer presenting as metastases to testis [20]. The exact mechanism of spread is unknown but many theories have been suggested. Since most cases of testicular metastases presented as a hydrocele, it is proposed that there may be microscopic channels of communications present between the peritoneum and testes. Other theories include retrograde venous and lymphatic extension, direct invasion, and arterial embolism [21]. In our case, our patient did not present primarily with testicular symptoms neither did he have hydrocele. He had a testicular nodule which was diagnosed to be metastatic adenocarcinoma on USG-guided needle biopsy.

Surgery is the only effective modality to cure patients with localized CRCs. However, for patients with cancer staging ≥ III, adjuvant chemotherapy is important to eradicate micrometastases, thereby reducing disease recurrence and increasing the cure rate [22].

Statistics show a higher incidence of unresectable residual disease and a higher metastasis rate in childhood CRCs [23]. Patients with unresectable tumors should undergo only biopsy and neoadjuvant chemotherapy, with or without radiation therapy.

The results of many series indicated that the overall survival of the patient depends on the complete surgical resection of the tumor and the radical resection of lymph nodes [24]; thus, it is the goal of surgery. However, only 40–69% of pediatric patients are candidates for curative resection, a much lower number than in adults (http://emedicine.medscape.com/article/993370-overview#a6). Debulking is of little benefit for patients with extensive metastatic disease. Occasionally, resections of bulky tumors or metastases offer palliation (http://emedicine.medscape.com/article/993370-overview#a6).

Predictors of poor prognosis, apart from the stage of the disease, are incomplete resection, mucinous histology, proportion of signet ring cells > 10%, and absence of an *in situ* component [22, 23]. In spite of all the advances in diagnosis and treatments, an overall 5-year survival is around 75% in adult patients but only around 51% in the pediatric population [22].

## Conclusions

CRC in the pediatric age group along with testicular metastasis is rare. It tends to be very aggressive and presents at a very advanced stage in pediatric patients, due to which it has a very poor prognosis. Due to its rarity, screening is not recommended for individuals under the age of 50. Thus, to improve outcome, early diagnosis and treatment is paramount. For that to happen, awareness among pediatricians and surgeons needs to be created regarding pediatric CRC and its signs and symptoms.
